# Effects of water-aging for 6 months on the durability of a novel antimicrobial and protein-repellent dental bonding agent

**DOI:** 10.1038/s41368-018-0019-9

**Published:** 2018-06-21

**Authors:** Ning Zhang, Ke Zhang, Michael D. Weir, David J. Xu, Mark A. Reynolds, Yuxing Bai, Hockin H. K. Xu

**Affiliations:** 1 Department of Orthodontics, School of Stomatology, Capital Medical University, Beijing, China; 2 Biomatexrials & Tissue Engineering Division, Department of Endodontics, Periodontics and Prosthodontics, University of Maryland Dental School, Baltimore, MD USA; 3 Center for Stem Cell Biology & Regenerative Medicine, University of Maryland School of Medicine, Baltimore, MD USA; 4 Marlene and Stewart Greenebaum Cancer Center, University of Maryland School of Medicine, Baltimore, MD USA; 5 Department of Mechanical Engineering, University of Maryland, Baltimore County, MD USA

## Abstract

Biofilms at the tooth-restoration bonded interface can produce acids and cause recurrent caries. Recurrent caries is a primary reason for restoration failures. The objectives of this study were to synthesize a novel bioactive dental bonding agent containing dimethylaminohexadecyl methacrylate (DMAHDM) and 2-methacryloyloxyethyl phosphorylcholine (MPC) to inhibit biofilm formation at the tooth-restoration margin and to investigate the effects of water-aging for 6 months on the dentin bond strength and protein-repellent and antibacterial durability. A protein-repellent agent (MPC) and antibacterial agent (DMAHDM) were added to a Scotchbond multi-purpose (SBMP) primer and adhesive. Specimens were stored in water at 37 °C for 1, 30, 90, or 180 days (d). At the end of each time period, the dentin bond strength and protein-repellent and antibacterial properties were evaluated. Protein attachment onto resin specimens was measured by the micro-bicinchoninic acid approach. A dental plaque microcosm biofilm model was used to test the biofilm response. The SBMP + MPC + DMAHDM group showed no decline in dentin bond strength after water-aging for 6 months, which was significantly higher than that of the control (*P* < 0.05). The SBMP + MPC + DMAHDM group had protein adhesion that was only 1/20 of that of the SBMP control (*P* < 0.05). Incorporation of MPC and DMAHDM into SBMP provided a synergistic effect on biofilm reduction. The antibacterial effect and resistance to protein adsorption exhibited no decrease from 1 to 180 d (*P* > 0.1). In conclusion, a bonding agent with MPC and DMAHDM achieved a durable dentin bond strength and long-term resistance to proteins and oral bacteria. The novel dental bonding agent is promising for applications in preventive and restorative dentistry to reduce biofilm formation at the tooth-restoration margin.

## Tooth restoration: Better bonding to beat bacteria

A strong, long-lasting bonding agent developed by researchers in the US and China can protect tooth restorations from bacteria and proteins. Tooth restorations often fail due to recurrent decay which starts when bacteria invade the interface between the tooth and the restoration material, producing acids. To combat this problem, Ning Zhang at the University of Maryland, Baltimore, and Capital Medical University, Beijing, and co-workers infused the commercial Scotchbond Multi-Purpose primer and adhesive with two chemicals known to inhibit the formation of bacterial biofilms. The team stored bonded teeth in warm water for six months, after which they found no decline in the bond strength, or its resistance to oral proteins and bacteria. They hope that by incorporating the same protective chemicals into cements, composites and sealants, failure rates in tooth restorations could be reduced.

## Introduction

Recurrent caries at the tooth-restoration bonded interface is a frequent reason for restoration failure.^1,2^ Bonding agents are employed to bond the restorations to tooth tissues, and the tooth-restoration interface is reported to be the weak link because bacterial invasion along the interface due to microgaps and leakage can result in recurrent caries.^3–7^ Therefore, extensive work has been devoted to enhance the tooth-restoration adhesion.^8,9^ In addition to increasing the bond strength, another good way to reduce biofilm formation at the margins is to apply antibacterial bonding agents. Indeed, novel quaternary ammonium methacrylates (QAMs) have been produced and added into polymeric composites and adhesives to deter oral biofilms.^3,4,10–18^ The antibacterial activity is increased by increasing the QAM alkyl chain length (CL) from 5 to 16.^19,20^ A recent study produced dimethylaminohexadecyl methacrylate (DMAHDM) with a CL of 16, which had the strongest antibacterial property among the several QAMs tested.^20^

Another approach to hinder biofilm growth is to produce dental resins that can resist protein attachment. It is often difficult to obtain a complete seal at the bonded interface; indeed, previous studies found microgaps at tooth-restoration margins.^21,22^ The microleakage could further worsen because of fatigue stresses, thereby affecting the durability of the bonded interface.^23,24^ The microgaps tend to accumulate oral biofilms, which produce acids that cause secondary caries.^23,24^ Protein attachment onto the resin is a prerequisite for bacterial adhesion.^25,26^ Hence, if the resin can resist proteins, then it could deter bacteria attachment. 2-Methacryloyloxyethyl phosphorylcholine (MPC) is a methacrylate with a phospholipid polar group in the side chain, and MPC-based polymers have shown strong resistance to protein attachment.^27–29^ A number of commercial medical products using MPC have been established with the approval of the United States Food and Drug Administration.^30–32^ Recently, MPC was blended with DMAHDM to produce a dental bonding agent that greatly reduced protein adhesion and biofilm growth.^33^ The novel MPC-DMAHDM adhesive strongly decreased protein attachment and oral biofilm activity without reducing the dentin bond strength. However, the endurance over time of the antimicrobial and protein resistance for the bonding agent containing MPC and DMAHDM has not been investigated.

Accordingly, the aim of this study was to investigate, for the first time, the effects of water-aging for 6 months on (1) the dentin bond strength using bonding agent containing MPC and DMAHDM, and (2) the resistance to protein adhesion and antimicrobial endurance over time in water-aging. The following hypotheses were tested: (1) the new bonding agent including MPC and DMAHDM would show no loss in dentin bond strength during 6 months of water-aging; (2) the new bonding agent containing MPC and DMAHDM would not show a decrease in resistance to protein adhesion or bacteria-eradicating function in 6-month water-aging; (3) compared with the commercial control, MPC + DMAHDM would greatly reduce protein adhesion and biofilm viability, even after 6 months of water-aging.

## Results

**Fig. 1:**
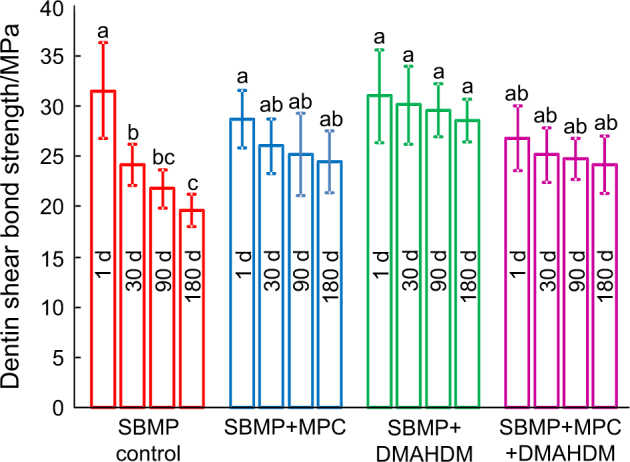
Dentin shear bond strength results (mean ± standard deviations; *n* = 10). The bond strength of SBMP control was significantly decreased during 180 d of water-aging (*P* < 0.05). By contrast, there was no significant strength loss with the bonding agents incorporating MPC and DMAHDM (*P* *>* 0.1). Bars with different letters indicate values that are significantly different from each other (*P* < 0.05)

The dentin shear bond strengths vs. water immersion time from 1 to 180 days (d) are plotted in Fig. 1 (mean ± standard deviations (SD); *n* = 10). The bond strength of Scotchbond multi-purpose (SBMP; 3M, St. Paul, MN), denoted SBMP control, significantly dropped during 180 d water immersion (*P* < 0.05). In the groups with MPC and DMAHDM, although there was a trend of decreasing bond strength with increasing time, the differences were not significant (*P* > 0.1). At 180 d, SBMP + MPC, SBMP + DMAHDM and SBMP + MPC + DMAHDM had greater dentin bond strength than SBMP control (*P* < 0.05).

**Fig. 2:**
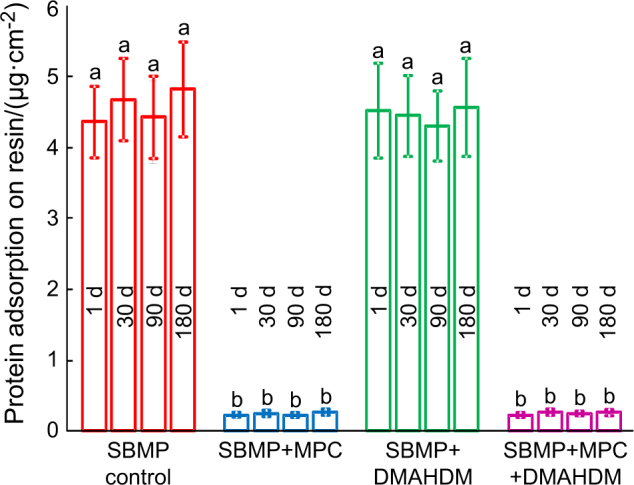
The amount of protein adsorption on cured resin disks (mean ± standard deviations; *n* = 6). SBMP + MPC + DMAHDM had protein adsorption that was nearly 1/20 that of SBMP control (*P* < 0.05). Its resistance to proteins did not decrease from 1 d to 180 d of water-aging. Values with dissimilar letters are significantly different (*P* *<* 0.05)

The results of protein adhesion onto the cured resin specimens are plotted in Fig. 2 (mean ± SD; *n* = 6). Adding MPC resisted the protein attachment (*P* *<* 0.05). Incorporation of DMAHDM had no effect on protein attachment (*P* *>* 0.1). The groups containing MPC had protein amounts that were approximately 1/20 that of SBMP control. Water-aging the resin specimens for 180 d prior to the protein test had no effect on protein attachment (*P* > 0.1), indicating that the resistance to protein adhesion by the resins containing MPC did not decline during water immersion.

**Fig. 3:**
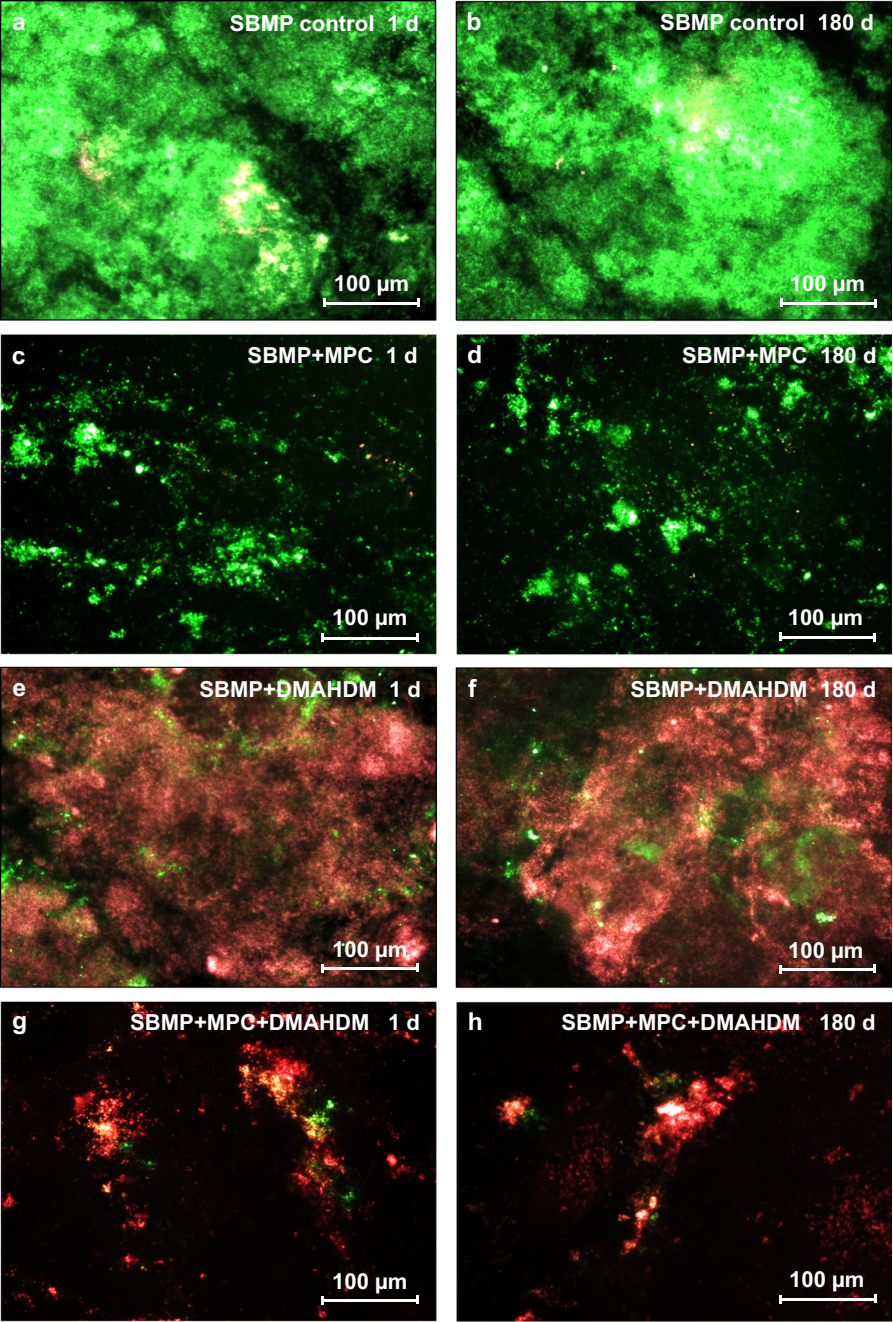
Representative live/dead staining images of biofilms grown for 2 days on resins. The group names and water-aging timers are indicated in each image. Live bacteria were stained green, and dead bacteria were stained red. SBMP + MPC decreased the bacterial coverage compared with SBMP control. SBMP + MPC + DMAHDM had less bacterial adhesion, and the biofilms consisted of primarily dead bacteria. Within each group, there was no noticeable difference between 1 d and 6 months. **a** SBMP control, water-aging for 1 d, **b** SBMP control, water-aging for 180 d, **c** SBMP+MPC, water-aging for 1 d, **d** SBMP+MPC, water-aging for 180 d, **e** SBMP+DMAHDM, water-aging for 1 d, **f** SBMP+DMAHDM, water-aging for 180 d, **g** SBMP+MPC+DMAHDM, water-aging for 1 d, **h** SBMP+MPC+DMAHDM, water-aging for 180 d.

Representative live/dead images of 2-d biofilms on resins are shown in Fig. 3. At 1 day, SBMP control was covered by live bacteria. By contrast, SBMP + MPC had much less bacterial adhesion, although the bacteria were mainly alive. SBMP + DMAHDM had generally compromised bacteria, and SBMP + MPC + DMAHDM had much less bacteria, which were predominantly compromised. After 180-d water immersion, the 2-d biofilm live/dead images were similar to those at 1 d, indicating that the resistance to proteins from MPC and the bacteria-killing ability from DMAHDM did not decline in water immersion.

**Fig. 4:**
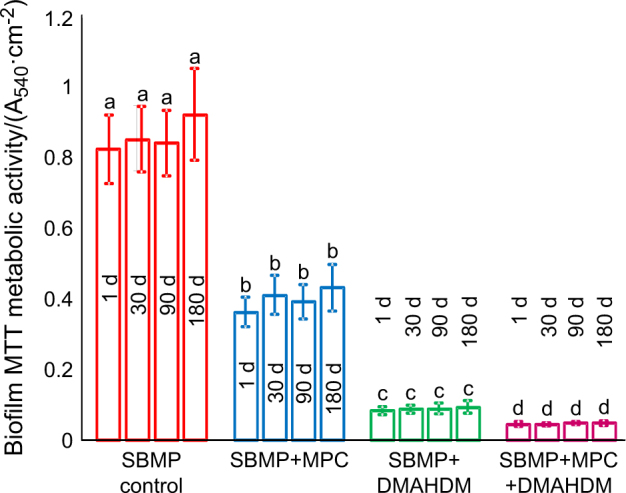
Biofilm MTT metabolic activity (mean ± standard deviations; *n* = 6). SBMP + MPC + DMAHDM had the least metabolic activity. Water-aging for 6 months did not reduce the antibacterial efficacy compared with 1 d (*P* > 0.1). Values with different letters are significantly different from each other (*P* *<* 0.05)

The metabolic activity of 2-d biofilms on resins is plotted in Fig. 4 (mean ± SD; *n* = 6). Incorporation of MPC or DMAHDM each diminished the metabolic activity of biofilms compared with SBMP (*P* < 0.05). Water-aging the resins for 6 months prior to the 3-(4,5-dimethylthiazol-2-yl)-2,5- diphenyltetrazolium bromide (MTT) test did not change the metabolic activity compared with 1 d (*P* > 0.1).

**Fig. 5:**
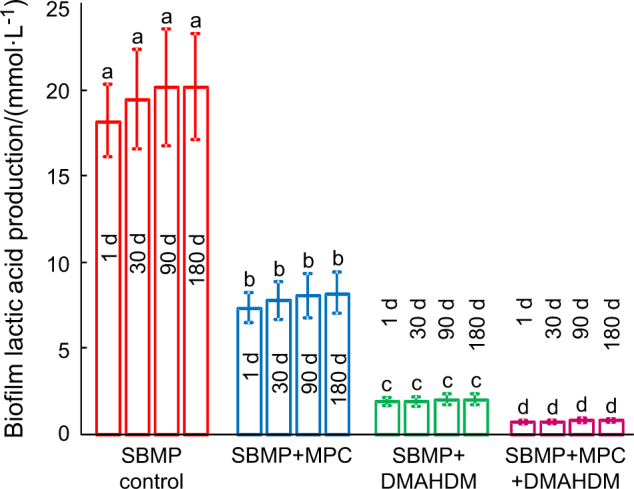
Biofilm lactic acid production (mean ± standard deviations;*n* = 6).Values with different letters are significantly different from each other (*P* *<* 0.05)

The lactic acid production of the 2-d oral biofilms on resins is plotted in Fig. 5 (mean ± SD; *n* = 6). For each group, water-aging the resins from 1 to 180 d did not affect the biofilm acid amount (*P* > 0.1). DMAHDM + MPC + DMAHDM had the least lactic acid from oral biofilms, which was nearly 1/20 that of SBMP control (*P* < 0.1).

**Fig. 6:**
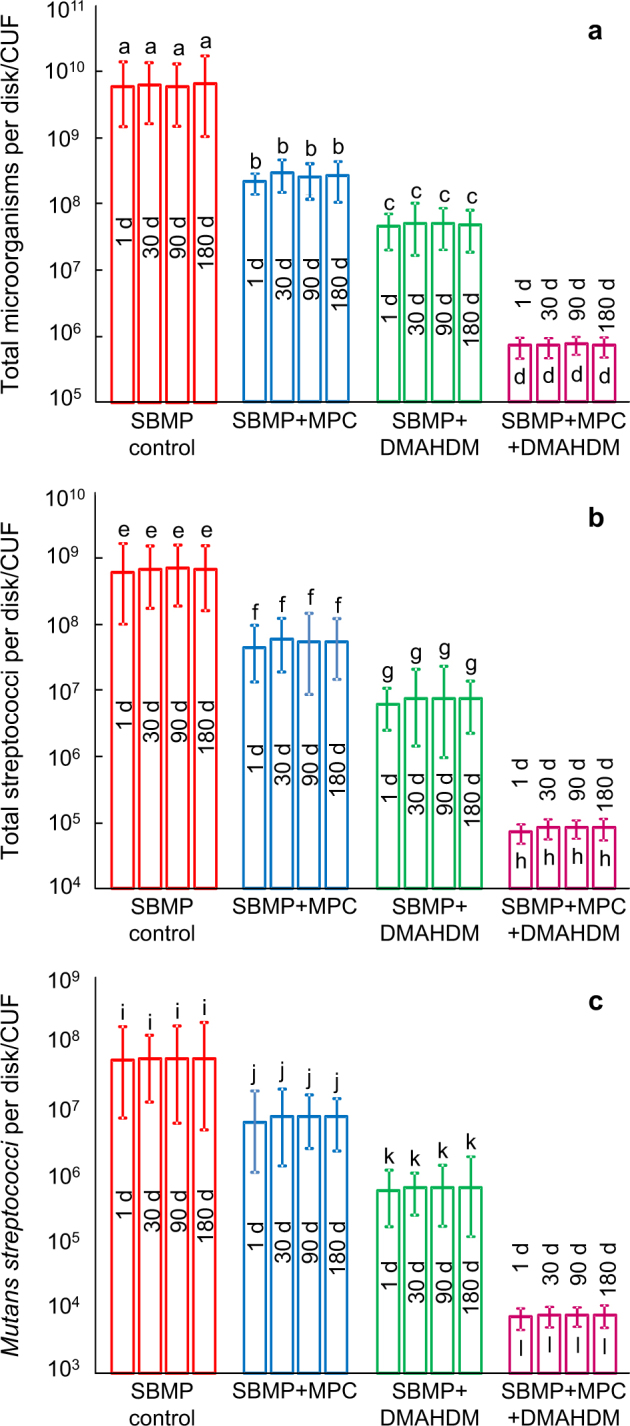
Colony-forming unit (CFU) counts for **a** total microorganisms, **b** total streptococci, and **c** *Streptococcus mutans* (mean ± standard deviations; *n* = 6). All three CFU counts on SBMP + MPC + DMAHDM were nearly four orders of magnitude less than that of SBMP control (*P* *<* 0.05). Within each group, there was no significant difference in CFU from before to after 6 months of water-aging (*P* > 0.1). In each plot, values with different letters are significantly different from each other (*P* *<* 0.05)

The colony-forming unit (CFU) counts of 2-d oral biofilms on resins are plotted in Fig. 6 for (a) total microorganisms, (b) total streptococci, and (c) *Streptococcus mutans* (mean ± SD; *n* = 6). In each group, there was no difference in CFU with water immersion from 1 to 180 d (*P* > 0.1). Adding MPC or DMAHDM into the resin decreased the CFU compared with SBMP control (*P* < 0.05). Using the MPC + DMAHDM combination recipe, SBMP + MPC + DMAHDM had much fewer CFUs than those using MPC or DMAHDM alone (*P* < 0.05). All three CFU counts on SBMP + MPC + DMAHDM were nearly four orders of magnitude (4 log) lower than SBMP control.

## Discussion

This study investigated the effects of water-aging for 6 months on the resistance to proteins and on the oral bacteria-eradicating ability of a novel bioactive MPC-DMAHDM dentin adhesive for the first time. The use of a combination agent (MPC + DMAHDM) was supported by the following four merits: (1) The use of the combination recipe in the bonding agent substantially decreased the oral plaque biofilm viability compared with that using MPC or DMAHDM alone. (2) The bonding agent using the combination agent demonstrated no loss in protein-repellent or anti-biofilm capability in 6 months of water-aging. (3) The use of the combination agent in the primer and adhesive did not lower the dentin bond strength. (4) The bonding agent using the combination recipe showed a significantly greater dentin bond strength at the end of the water-aging period than the commercial control.

DMAHDM is a quaternary ammonium monomer with an alkyl CL of 16, which exhibited the greatest antibacterial potency among a groups of QAMs tested in one study.^20^ The antibacterial mechanism of QAMs is that quaternary ammonium can lead to bacteria lysis by adhering to the cell membrane to produce cytoplasmic leakage. When the bacterial cell touches the QAM resin, the electric balance of the cell membrane is compromised, resulting in cell death.^34–36^ Regarding the endurance of the antibacterial ability, QAMs in resins have achieved long-lasting antibacterial ability because the QAM copolymerizes with the resin by forming a covalent bond with the polymer network.^3,4,12^ Therefore, the QAM is immobilized in the resin and is not lost over time, thus imparting a durable antibacterial capability.^3,4,12^ Indeed, a recent report on a bonding agent containing dimethylaminododecyl methacrylate (DMADDM) demonstrated that the antibacterial properties had no decrease after water immersion from 1 d to 6 months.^37^ This is consistent with the present study on DMAHDM, which showed no significant decrease in antibacterial efficacy after 6 months of submersion in water.

Another essential component is MPC, which is very hydrophilic.^27^ There is ample free water but no bound water in the hydrated MPC polymer.^29,38^ The ample free water around the phosphorylcholine group makes it hard for proteins to attach. Hence, the MPC polymer can deter protein and bacterial attachment.^29,38^ Regarding the durability of protein deterrence, MPC contains reactive methacrylate groups, which can copolymerize and covalently bond with the resin matrix when the material is light cured.^39^ MPC can copolymerize with acrylic resin through strong C-C bonding, offering long-lasting resistance to protein adhesion.^39^ Furthermore, previous reports showed that an MPC-containing surface layer was resistant to mechanical stresses.^38,40^ In the present study, MPC was blended into the resin throughout the volume and not merely on the surface. This characteristic may provide a durable protein-repellent effect in vivo, and further studies should investigate the long-lasting protein-repellent ability of this novel bioactive bonding agent after brushing and chewing tests. The SBMP contained 2-hydroxyethyl methacrylate (HEMA) and a copolymer of acrylic/itaconic acids, which could copolymerize with MPC, thus offering long-lasting resistance to protein attachment. Incorporation of MPC into resin strongly reduced the amount of protein adhesion, and there was no difference between 1 d and 6 months of water immersion, indicating excellent endurance of its protein resistance.

In addition, the adhesion of salivary proteins on cationic antimicrobial surfaces can reduce the antibacterial potency of QAMs because the protein film inhibits the direct contact between bacteria and the antibacterial surface, thus reducing the “contact-inhibition” efficacy.^34,36,41,42^ However, MPC can strongly decrease the protein attachment. MPC can minimize the saliva-derived protein film coatings on the cationic antibacterial surfaces, thereby increasing the direct contact between bacteria and the antimicrobial surface, which in turn boosted the antibacterial effect of DMAHDM in this study. Indeed, the results in Figures 3–6 confirm that SBMP + MPC + DMAHDM had stronger antibacterial effects than using DMAHDM without MPC. Therefore, the use of the combination agent, MPC + DMAHDM, produced a synergistic effect owing to MPC’s boosting of direct bacteria–resin contact, which stimulated the antibacterial efficacy of DMAHDM.

Another essential requirement of dental bonding agents is the endurance of dentin bond strength.^43^ Water adsorption is inescapable in the oral environment because of the hydroxyl groups in the bonding agent systems.^44^ However, this may lead to hydrolysis of the hydrophilic resin.^45,46^ In addition, the host-derived matrix metalloproteinases (MMPs) could lead to dissolution of the exposed collagen fibrils in the hybrid layer.^47,48^ The dissolution of collagen may increase the water content, resulting in further collagen degradation and originating the deterioration of the dentin bond.^49^ Furthermore, several uncured monomers and break-down products of the tooth-restoration margin can diffuse out, thus decreasing the bond strength. QAMs exhibit beneficial MMP inhibitory and anti-enzyme properties.^50^ In the present study, the dentin shear bond strength of the control group markedly decreased after prolonged water immersion. By contrast, DMAHDM-containing bonding agents showed no significant reduction in bond strength after prolonged water immersion. This was likely because DMAHDM copolymerized with the adhesive and did not diffuse out, thus exerting long-lasting antimicrobial effects. The MPC group also exhibited no significant loss in dentin bond strength at 180 d, possibly because MPC may have an anti-MMP ability similar to QAMs. This may be because MPC contains a quaternary ammonium group,^27,51^ which is analogous to that in QAMs. In addition, MPC contains a negatively charged phosphate group.^27,51^ Therefore, this chemical structure may allow MPC to apply electrostatic interactions and vary the configuration of the active sites of MMPs, yielding suppressive effects on MMPs.^50^

Potential applications of the novel bioactive bonding agent containing MPC and DMAHDM include the bonding of composite restorations in tooth cavities to reduce biofilms and decrease acid production. The MPC and DMAHDM combination agent is also promising for incorporation into composites, cements, sealants and various other resin systems. For example, the use of MPC and DMAHDM dual agents in class V restorations with subgingival margins may be able to inhibit subgingival biofilm formation. Microleakage at the tooth-restoration margin could lead to recurrent caries, and microleakage could further worsen after water-aging for long periods of time. Further studies are needed to use this novel bioactive bonding agent in bond restorations to tooth tissues and investigate the amount of microleakage at the tooth-restoration interface after long-term aging under in vivo-mimicking conditions.

## Materials and methods

### Incorporation of MPC into bonding agent

SBMP (3M, St. Paul, MN) was used as a commercial control. Its adhesive comprises 60%–70% bisphenol A diglycidyl methacrylate (BisGMA) and 30%–40% HEMA, tertiary amines and a photo-initiator. Its primer has 35%–45% HEMA, 10%–20% a copolymer of acrylic and itaconic acids, and 40%–50% water. MPC was obtained from Sigma-Aldrich (St. Louis, MO), which was produced using a previously reported method.^27^ MPC powder was mixed with SBMP primer at the ratio of MPC/(SBMP primer + MPC) of 7.5% by mass. The 7.5% was chosen according to a previous report.^33^ The primer was blended magnetically with a bar at a speed of 150 r ·min^−1^ (Bellco Glass, Vineland, NJ) for 24 h to completely dissolve MPC in SBMP primer.^33^ Similarly, 7.5% MPC was also blended into SBMP adhesive.

### Incorporation of DMAHDM into bonding agent

DMAHDM was produced using a modified Menschutkin reaction approach following previous experiments.^20,52,53^ 2-(Dimethylamino)ethyl methacrylate (DMAEMA, Sigma-Aldrich) at 10 mmol and 1-bromohexadecane (BHD, TCI America, Port-land, OR) at 10 mmol were mixed with ethanol at 3 × *g* in a scintillation vial. The vial was stirred at a temperature of 70 °C for 1 d. When the solvent was evaporated, DMAHDM was produced, which is a viscous liquid that is colorless.^20,52,53^ DMAHDM at 5% mass fraction was blended into SBMP primer and adhesive. Mass fractions >5% were not used because our preliminary study showed that they lowered the dentin bond strength when 7.5% MPC was also blended into the resin. The following four bonding systems were investigated:

1. SBMP primer and adhesive control (termed “SBMP control”);
2. 92.5% SBMP primer + 7.5% MPC, 92.5% SBMP adhesive + 7.5% MPC (termed “SBMP + MPC”);
3. 95% SBMP primer + 5% DMAHDM, 95% SBMP adhesive + 5% DMAHDM (termed “SBMP + DMAHDM”);
4. 87.5% SBMP primer + 7.5% MPC + 5% DMAHDM, 87.5% SBMP adhesive + 7.5% MPC + 5% DMAHDM (termed “SBMP + MPC + DMAHDM”).

To make resin disks, the cover of a sterile 96-well plate was used as the mold following previous experiments.^20^ Ten microliters of a primer was placed in the bottom of each dent, which was dried with air. Then, 20 µL of adhesive was applied to the dent and photo-polymerized for 30 s (Optilux-VCL401, Demetron, Danbury, CT, USA) by covering with a Mylar sheet. This produced a cured disk of 8 mm in diameter and 0.5 mm in thickness. The disks were submerged in 200 mL of distilled water and agitated magnetically with a bar at a speed of 100 r ·min^−1^ for 1 h to remove any monomers that were not polymerized, as in a previous report.^10^

### Dentin shear bond testing

Extracted healthy human molars were sawed to cut off the crowns (Isomet, Buehler, Lake Bluff, IL), then ground using 320-grit silicon carbide abrasive paper to remove occlusal enamel.^17,18^ Dentin was acid-etched for 15 s and washed with water.^43^ A primer was applied, and the solvent was removed with an air stream. An adhesive was applied and photo-cured for 10 s. To test the bond strength, a stainless-steel iris (central opening diameter = 4 mm, height = 1.5 mm) was placed against the etched dentin. A composite paste was filled into the central opening (TPH, Caulk/Dentsply, Milford, DE), which was light cured for 60 s.^43^ A chisel was placed to be parallel to the bonded interface, and force was applied using a Universal Testing Machine (MTS, Eden Prairie, MN) at a displacement rate of 0.5 mm ·min^-1^. The force was increased until the composite-dentin bond fractured. Dentin shear bond strength S_D_ was calculated using the following equation: S_D_ = 4 P/(πd^2^) where P is the load at fracture, and d is the diameter of the composite.^17,43^ Ten teeth were tested for each group.

### Water-aging

Samples for dentin bond strength and protein-repellent and antibacterial properties were submerged in distilled water at 37 °C for 1, 30, 90, or 180 d. Each group was placed in 200 mL of water in a sealed polyethylene container, following previous experiments.^54,55^ Every week, the water was changed once. At the end of each time period, the dentin shear bond strength of the samples was measured as described above in Section 2.3 (*n* = 10 for each group at each time point). The resin disks of 8 mm in diameter and 0.5 mm in thickness for protein attachment and antibacterial experiments (*n* = 6 for each group at each time point) were sterilized with ethylene oxide (Anprolene AN 74i, Andersen, Haw River, NC) and de-gassed for 7 d.^20,43^

### Measurement of protein adsorption

Protein attachment on resin disks of 8 mm in diameter and 0.5 mm in thickness (*n* = 6, for each group at each time point) was measured via a micro-bicinchoninic acid (BCA) approach.^33,39,56^ Each sample was placed for 2 h in phosphate-buffered saline (PBS). Samples were submerged at 37 °C for 2 h in bovine serum albumin (BSA) solution (Sigma-Aldrich). The protein solution included BSA at 4.5 g ·L^−1^ concentration, according to a former report.^57^ The disks were then washed for 5 min with fresh PBS by stirring at 300 r· min^−1^ (Bellco Glass, Vineland, NJ). The attached proteins were removed in sodium dodecyl sulfate (SDS) at 1% in PBS via sonicating for 20 min. BSA concentration in the SDS solution was evaluated using a protein analysis kit (micro BCA, Fisher Scientific, Pittsburgh, PA).^33,39,56^ Briefly, a mixture 25 µL of the SDS solution with 200 µL of the BCA working reagent in a 96-well plate was incubated at 60 °C for 30 min.^33,39,56^ Then, the 96-well plate was cooled to room temperature, and the absorbance at 562 nm was measured via a microplate reader (SpectraMax M5, Molecular Devices, Sunnyvale, CA). Standard curves were prepared using BSA standard. From the concentration of protein, the amount of protein on the sample surfaces was determined.^33,39,56^

### Dental plaque microcosm biofilm formation and live/dead assay

Ten adults donated saliva for use as the biofilm inoculum, following a protocol approved by the University of Maryland Institutional Review Board. The donors had natural dentition, had no active caries, had no periopathology, and had not used antibiotics in the past three months.^17,18^ The donor did not brush teeth for 24 h and had no food/drink intake in the past 2 h before donating saliva. The collected saliva was diluted in sterile glycerol to a concentration of 70% and stored at −80 °C.^57^ The saliva-glycerol stock was added, at 1:50 final dilution, to a growth medium as an oral bacteria inoculum. The growth medium comprised mucin (type II, porcine, gastric) at a concentration of 2.5 g· L^−1^; bacteriological peptone, 2.0 g· L^−1^; tryptone, 2.0 g· L^−1^; yeast extract, 1.0 g· L^−1^; NaCl, 0.35 g ·L^−1^, KCl, 0.2 g· L^−1^; CaCl_2_, 0.2 g· L^−1^; cysteine hydrochloride, 0.1 g· L^−1^; hemin, 0.001 g· L^−1^; vitamin K1, 0.000 2 g· L^−1^, at pH 7.^58^ Into each well of 24-well plates with a resin disk, 1.5 mL of inoculum was poured and cultured at 37 °C in 5% CO_2_ for 8 h. Then, the disks were moved to new 24-well plates filled with new culture medium. After 16 h, the disks were moved to new 24-well plates with new culture medium and cultured for 24 h. This totaled 48 h of culture, which was enough to form oral plaque microcosm biofilms on resins in vitro, as shown in previous experiments.^17,18,57^

A BacLight live/dead kit (Molecular Probes, Eugene, OR) was used to stain samples (resin disks of 8 mm in diameter and 0.5 mm in thickness) with 2-d biofilms (*n* = 6, for each group at each time point).^17,18^ When live bacteria were stained with Syto 9, a green fluorescence was produced. When bacteria with compromised membranes were stained with propidium iodide, a red fluorescence was produced. An epifluorescence microscope (Eclipse TE2000-S, Nikon, Melville, NY) was used to examine the stained samples.

### MTT metabolic assay

Resin disks of 8 mm in diameter and 0.5 mm in thickness (*n* = 6, for each group at each time point) were cultured for 2 d to form oral microcosm biofilms as described above. They were placed to a new 24-well plate for the MTT assay.^17,18^ To each well, 1 mL MTT was added and incubated for 1 h. Disks were moved to a new 24-well plate, and to solubilize formazan, 1 mL of dimethyl sulfoxide (DMSO) was added to each well. The plate was incubated for 20 min in a darkroom. A microplate reader (SpectraMax M5, Molecular Devices, Sunnyvale, CA) was used to analyze the absorbance at 540 nm for 200 µL of DMSO solution harvested from each well.^17,18^

### Lactic acid production and CFU counts

Resin disks of 8 mm in diameter and 0.5 mm in thickness (*n* = 6, for each group at each time point) with 2-d biofilms were washed with cysteine peptone water (CPW) to detach loose bacteria.^17^ The disks were put in 24-well plates containing buffered peptone water (BPW). Then, 0.2% sucrose was added, and the samples were cultured at 37 °C in 5% CO_2_ for 3 h, whereas the oral biofilms generated acids.^17^ The BPW solutions were then stored for lactate analysis. Lactate concentrations in the BPW solutions were evaluated via an enzymatic (lactate dehydrogenase) method, following a previous report, with standard curves constructed using a lactic acid standard (Supelco, Bellefonte, PA).^17^ The aforementioned microplate reader was used to measure the absorbance at 340 nm for the collected BPW solutions.

To measure the CFU, resin disks of 8 mm in diameter and 0.5 mm in thickness (*n* = 6, for each group at each time point) with 2-d biofilms were moved to tubes with 2 mL of CPW, and sonication and vortexing (Fisher, Pittsburgh, PA) were performed to harvest the biofilms from the disks.^17,18,20^ To analyze the CFU counts of the oral biofilms, three types of agar plates were prepared. The first were tryptic soy blood agar culture plates to determine the total microorganisms.^58^ The second were mitis salivarius agar (MSA) culture plates with 15% sucrose for determining the total streptococci.^59^ The third were MSA agar plates containing 0.2 units of bacitracin per mL for evaluating the *Streptococcus mutans*.^58^ The bacterial suspensions were serially diluted, spread onto agar plates and incubated at 37 °C in 5% CO_2_ for 24 h. The number of colonies that grew was counted and used, along with the dilution factor, to calculate total CFU counts on each disk.^17,18,20^

### Statistical analysis

All data were first checked for normal distribution with the Kolmogorov–Smirnov test and tested for homogeneity using Levene’s test. For the protein-repellent experiment, MTT metabolic assay and acid production assay, inter-group differences were estimated using analysis of variance (ANOVA) for factorial models, and individual groups were compared using Fisher’s protected least-significant difference test. For CFU, the values were first transformed by log_10_ to normalize the data distribution, and then ANOVA and Fisher’s protected least-significant difference test were performed. Statistical analyses were performed by SPSS 13.0 software (SPSS Inc., Chicago, IL) at a significance level of *P* < 0.05.

## Conclusions

A new bonding agent containing both MPC and DMAHDM had significantly greater dentin bond strength than commercial control after water-aging for 6 months. In addition, the MPC + DMAHDM bonding agent markedly decreased protein attachment and oral biofilm viability. There was no significant decrease in the protein-repellent or anti-biofilm effect from 1 d to 6 months. The bonding agent using MPC + DMAHDM is promising for dental bonding to inhibit biofilm formation and reduce acid production.
